# Case Report: The Monogenic Familial Steroid-Resistant Nephrotic Syndrome Caused by a Novel Missense Mutation of *NPHS2* Gene A593C in a Chinese Family

**DOI:** 10.3389/fped.2021.692727

**Published:** 2021-09-23

**Authors:** Ling Bai, Jing Zhuang, Changrong Zhang, Chen Lu, Xuefei Tian, Hong Jiang

**Affiliations:** ^1^Department of Nephrology and Rheumatology, Children's Hospital of Xinjiang Uygur Autonomous Region, Urumqi, China; ^2^Division of Nephrology, Department of Internal Medicine, People's Hospital of Xinjiang Uygur Autonomous Region, Urumqi, China; ^3^Division of Nephrology, Department of Internal Medicine, The First Affiliated Hospital of Xinjiang Medical University, Urumqi, China; ^4^Section of Nephrology, Department of Internal Medicine, Yale University School of Medicine, New Haven, CT, United States

**Keywords:** steroid-resistant nephrotic syndrome, mutation, *NPHS2*, genetic testing, focal segmental glomerulosclerosis

## Abstract

**Background:** Pathogenic variants in the *NPHS2* gene encoding podocin in kidney podocytes are associated with autosomal recessive steroid-resistant nephrotic syndrome (SRNS) by disrupting podocyte function and the integrity of the glomerular filtration barrier. The outcome is generally poor by progressing into end-stage kidney disease (ESKD). With the help of gene diagnostics, we can further understand the role of podocin of podocytes in the development and progression of SRNS. However, the pathological mutation of *NPHS2* and clinical relevance remain further elusive.

**Case Presentation:** Two siblings, a 15-year-old girl and her 10-year-old younger brother from a consanguineous Chinese family, presented with nephrotic syndrome. Both of them developed progressive proteinuria starting from the 5-year-old of age. The renal pathological lesions for them revealed focal segmental glomerulosclerosis (FSGS). There was no response to the glucocorticoid, calcineurin inhibitors, and rituximab treatment. The female affected patient received the hemodialysis treatment due to ESKD in June 2020; the male patient was still in follow-up presenting with SRNS. The mutational screening of the two patients and their parents using Trio whole-exome sequencing showed the *NPHS2* gene *de novo* missense mutation in exon 5 (A593C), for which the two siblings were homozygous and their parents confirmed heterozygous asymptomatic carriers. No other SRNS-related gene variants with the SRNS were determined.

**Conclusion:** Pathological gene variants screening in children clinically suspected with SRNS might be helpful in the diagnosis as well as appropriate decisions on treatment strategies and prediction of prognosis.

## Introduction

Nephrotic syndrome (NS) is a common glomerular disease characterized by massive proteinuria, hypoalbuminemia, hyperlipidemia, and edemas. NS can be classified into hereditary, primary, and secondary types based on their etiologies. Meanwhile, the NS can be clinically divided into two types depending on the response of patients to the steroid therapy, including the steroid-sensitive NS (SSNS) and steroid-resistant NS (SRNS) (1, 2). Podocyte plays a crucial role in maintaining the integrity and stability of the glomerular filtration barrier (3); in children-onset and early adult-onset SRNS, mutation of more than 50 genes in the podocyte have been associated with two-third of these patients, especially nephrin (*NPHS1*), and podocin (*NPHS2*) (4–6). The SRNS is the second most cause leading to end-stage kidney disease (ESKD) in children.

The *NPHS2* (OMIM number 604766) is localized on chromosome 1q25-q31, it was first reported and mapped by linkage analysis in families with autosomal recessive SRNS in 2000, in which 10 different *NPHS2* mutations were identified (7). *NPHS2* comprises of 8 exons and encodes the 42 KD protein podocin with a 383-amino acid in the podocyte (8). Podocin localizes at the slit diaphragm in podocyte and is required for the maintenance of the integrity of the glomerular filtration barrier by interacting with nephrin, CD2AP, and TRPC6 et al., thereby orchestrating the mechanosensation signaling, cytoskeleton organization, and cell survival (9). The mutation of *NPHS2* has been reported to occur in 13% of patients that manifested with SRNS before 25 years of age and accounted for the 34% of all 27 common genes in podocytes causing SRNS (10). To date, many novel *NPHS2* gene mutations have been discovered and more genotype-phenotype correlations have been published, such as deletion c.988_989delCT, c.725C>T, c622G>A and c.928G>A et al. (11–13). With the advent of the latest next gene screening technology and the development of molecular genome database platforms, more and more pathogenic genetic mutations contributing to pediatric SRNS have been identified in recent years (14–16). In this study, we presented a case of two siblings from a consanguineous Chinese family with *NPHS2* mutation, whose renal histology showed focal segmental glomerulosclerosis (FSGS) and had no response to glucocorticoid, calcineurin inhibitors, and rituximab therapy. To the best of our knowledge, this is the first case report of the novel missense mutation of *NPHS2* in exon 5 (A593C) contributing to the SRNS. Our reports may help to further understand the pathogenesis of hereditary glomerulonephropathy caused by *NPHS2* mutation in podocytes, may help the clinicians to choose the proper treatment strategies and genetic and reproductive counseling.

## Case Presentation

**Figure 1:**
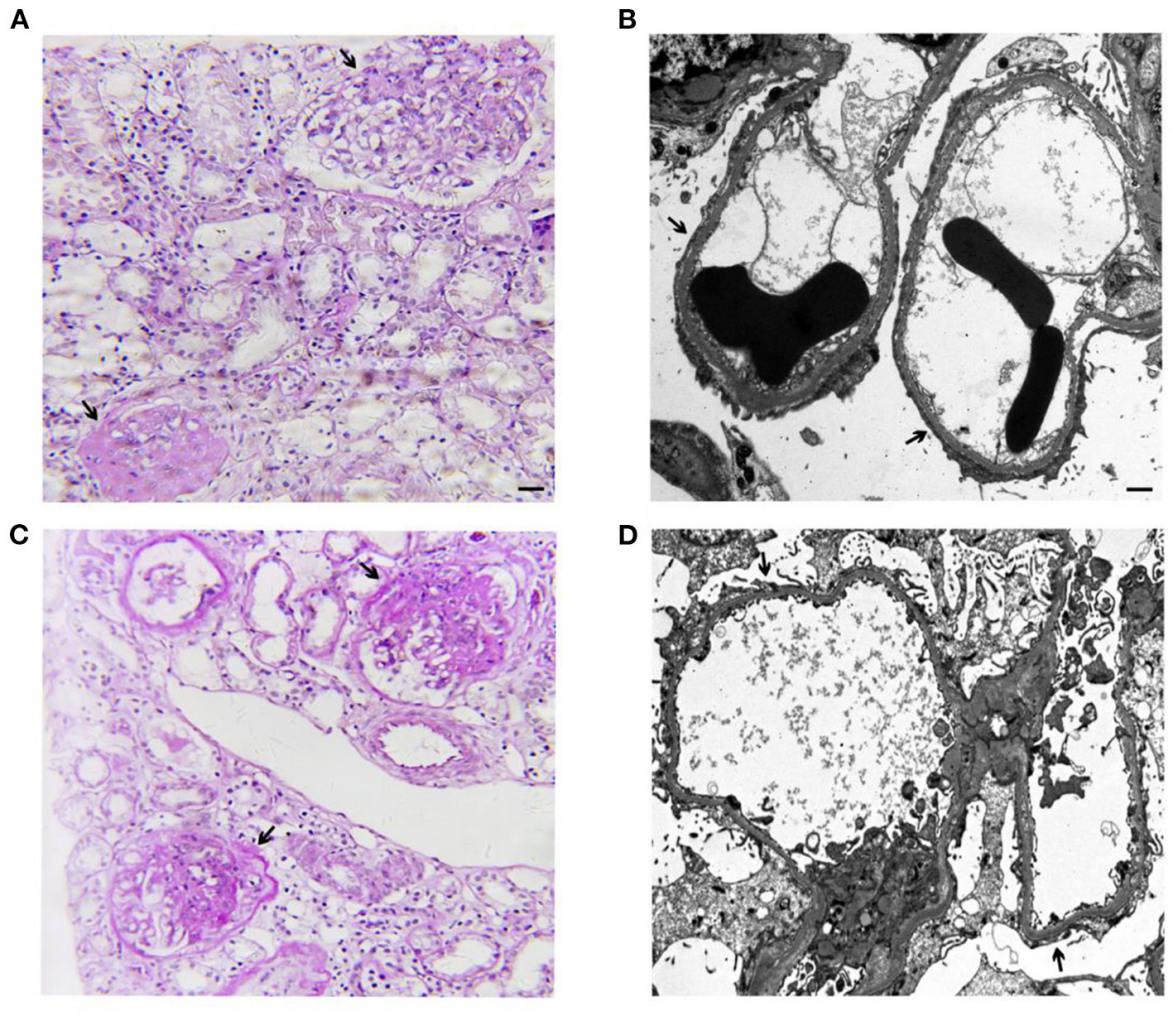
Representative images of light microscopy and transmission electron microscopy (TEM) from the kidney biopsies of the elder sister at age 12 **(A,B)** and the younger brother at age 8 **(C,D)**. **(A)** Global or segmental glomerulosclerosis (arrow) in 23 of 38 glomeruli (Periodic Acid-Schiff staining. Scale bar: 50 μm). Immunofluorescence microscopy revealed negative for IgG, IgA, IgM, C3, C1q, kappa light chain, and lambda light chain in 2 glomeruli. **(B)** Representative TEM showed diffuse foot process effacement of the podocyte (arrow), partial irregularity of the glomerular basement membrane, foam cells, and no subendothelial and mesangial dense deposits (Scale bar: 1 μm). **(C)** Global or segmental glomerulosclerosis (arrow) in 7 of 33 glomeruli (Periodic Acid-Schiff staining. Scale bar: 50 μm). Immunofluorescence microscopy revealed mesangial IgM deposits (++) negative and negative results for IgG, IgA, C3, C1q, kappa light chain, and lambda light chain in 2 glomeruli. **(D)** Representative TEM showed diffuse foot process effacement of the podocyte (arrow), partial irregularity of the glomerular basement membrane, foam cells, and no subendothelial and mesangial dense deposits (Scale bar: 1 μm).

A 15-year-old Chinese girl was first diagnosed with proteinuria (2+) during a regular check-up at the age of 5 years old in 2011 but remained untreated until the age of 12 years old in 2018 when she was diagnosed with nephrotic syndrome. The child was transferred to our hospital for further evaluation. There was no abnormal birth and past medical history. Physical examination revealed edema, no skin rash or arthralgia, or gross hematuria. Laboratory studies indicated nephrotic syndrome without hematuria. Blood pressure and serum creatinine levels were normal. Screening of serum and urine from the patient excluded the presence of systemic lupus erythematosus (SLE), antineutrophil cytoplasmic antibody-associated vasculitis (AAV) et al. Since the proteinuria and hypoalbuminemia were resistant to the steroid treatment, prednisone 2 mg/kg per day for 8 weeks, a renal biopsy for her at the age of 12 years old was performed showing FSGS (Figures 1A,B). Based on pathological lesion assessment, intravenous methylprednisolone pulse, prednisone plus FK506 (blood trough levels 1.7–2.5 ng/ml), cyclosporine A, and rituximab were administered for 2 years. However, the patient presented persistent nephrotic syndrome and progressive renal failure. At the age of 14 years old in 2020, the patient started on hemodialysis because of ESKD.

Her sibling, a 10-year old Chinese boy, was diagnosed with proteinuria at the age of 5 years old in 2016 during a regular check-up. He was diagnosed with nephrotic syndrome at the age of 8 years old in 2019 and he was admitted to the hospital because of the exacerbating edema in the abdomen. There was no abnormal birth and past medical history. Screening of serum and urine from the patient excluded the presence of SLE, AAV et al. The renal biopsy of the patient at the age of 8 years old was carried out revealing FSGS (Figures 1C,D). The proteinuria and hypoalbuminemia were unresponsive to the treatment with prednisone (2 mg/kg per day) and cyclosporin A for 8 weeks, following by the treatment with several pulses of cyclophosphamide and methylprednisone. The patient is presently on non-immunosuppressive antiproteinuric treatment including the optimized renin-angiotensin system (RAS) blockade under the outpatient follow-up, with a relatively stable kidney function determined by serum creatinine (16.5–19.2 μmol/L).

There was a consanguineous family history of father and mother being first cousins for the two siblings, the parents do not present any symptoms of kidney disease.

**Figure 2:**
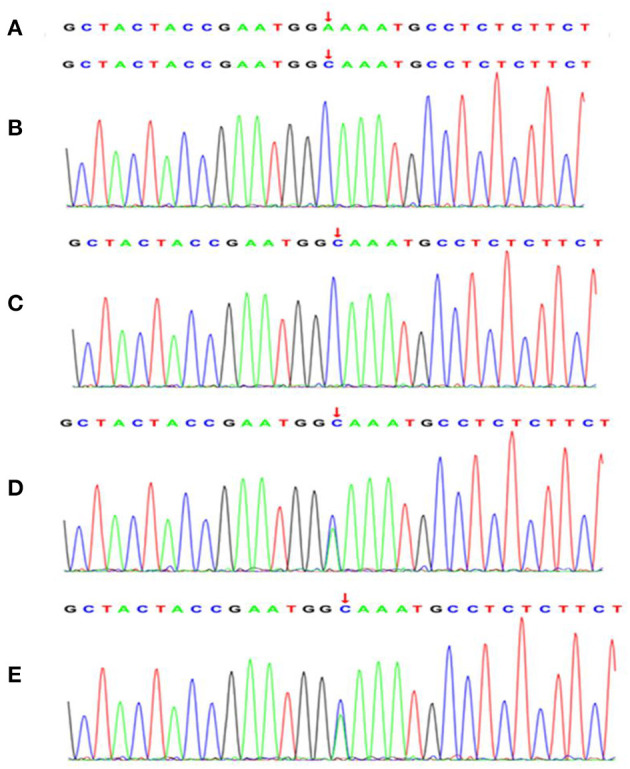
A novel missense mutation in exon 5 (A593C) of the *NPHS2* gene was identified by Trio whole-exome sequencing in the affected individuals and family members. **(A)** normal control sequence. The affected elder sister **(B)** and the affected younger brother **(C)** carried the homozygous missense mutation in exon 5 (A593C) of the *NPHS2* gene. The father **(D)** and the mother **(E)** of the affected patients revealed the heterozygous missense mutation in exon 5 (A593C) of the *NPHS2* gene. The arrows indicate mutant positions.

Because of the SRNS of these two siblings and the consanguineous family history, genetic testing was recommended. All patients and their parents gave their consent for inclusion in this study. Genetic analysis for these two siblings and their parents was carried out by next-generation sequencing (NGS)-based Trio whole-exome sequencing (WES) (Chigene translational medical research center Co. Ltd., Beijing, China). Briefly, genomic DNA was isolated from peripheral blood leukocytes with EDTA as the anticoagulant, sequences of exons were captured using IDT probes (IDT xGen Exome research pane 1 v1.0) and sequenced using NGS sequencer Genome analyzer (NovaSeq 6000, PE150, Illumina). SRNS-related target variants identified by Trio WES were verified by Sanger sequencing (ABI 3730 DNA Analyzer). This revealed a novel missense mutation in exon 5 c.593 A>C (p. E198A) of the *NPHS2* gene, the two siblings were found to be homozygous and their parents were both heterozygous for this mutation. The result of minor allele frequency (MAF) of the *NPHS2* c.593 A>C missense mutation was <0.0005 in the general population, a low-frequency mutation; the missense mutation results in the alanine instead of the glutamic acid in the 198th position of the amino acid sequence of the wild-type podocin (NM_014625). The missense mutation was not found in the normal control and was listed as “likely pathogenic” by ACMG (The American College of Medical Genetics and Genomics) 2015 guideline after analyzing the genotype-phenotype associated diseases database and genetic diagnostic platform. Novel missense mutation variants identified in this study were shown in Figure 2. The family cosegregation analysis of the phenotype-genotype for these two patients and their parents indicated the autosomal recessive inheritance type.

## Discussion

In this study, we described two siblings with SRNS who carried a novel homozygous missense mutation in exon 5 c.593 A>C (p. E198A) in the *NPHS2* gene, which encodes the podocin in the podocyte. No other mutations were found in the *NPHS2* gene or other SRNS-related genes such as *NPHS1, WT1, FN1, KANK2, BMP4*, and *PLOD1* et al. Their father and mother carried the same heterozygous missense mutation as the siblings. The parents showed no proteinuria, hematuria, or other abnormalities. It suggests that a missense mutation on both alleles in exon 5 c.593 A>C of the *NPHS2* gene is required for the development and progression of SRNS. The percentage of SNRS attributing to *NPHS2* variants varies from 12.7 to 29.5% (10, 17, 18). The *NPHS2* mutation usually presents the autosomal recessive form of hereditary SRNS, the phenotype is less severe than that of the SRNS caused by the *NPHS1* variants, which is one of the most common mutations resulting in the hereditary podocytopathy in children, and it encodes the nephrin in the podocyte. The onset age of patients with *NPHS1* mutation is at birth to 3 months, while it is later than 3 months at the onset age for the patients with *NPHS2* mutation (10, 19). The two siblings reported here both presented with SRNS and pathological lesions of FSGS, both of them were found proteinuria at 5 years old. Unfortunately, there were no medical records of their urinary analysis results in the past although their parent said that the two siblings were normal in appearance. Both of the two siblings failed to respond to steroid therapy and immunosuppressive agents, which did not have an impact on the etiological target of hereditary podocytopathy. It is worth noting, the longtime treatment with steroids and immunosuppressive agents causes side effects of drugs and increases the financial burdens to the family. The elder sister was started on hemodialysis at 14 years old due to ESKD, and the younger brother is currently on symptomatic treatment.

The underlying missense mutation of the *NPHS2* gene of patients in our study localized in the exon 5 encoding for the central part of the podocin consisting of 68 amino acids (from 179th amino acid to 246th amino acid), which suggests this area is of major importance for the proper function of podocin (20). Besides this mutation, two variants in exon 5 of the *NPHS2* gene have been reported to cause the SRNS (p.R229Q and p.A242V) in the population of Caucasians and Europeans (2). The c.593 A>C mutation of the *NPHS2* gene results in the alanine instead of the glutamic acid in the 198^th^ position of the amino acid sequence of the podocin. Podocin is a membrane protein that is exclusively located in the slit diaphragm of the podocyte in the kidney (21). Podocin plays a critical role in the stability of the slit diaphragm and the maintenance of the integrity of the glomerular filtration barrier by forming a protein complex through co-localizing with other proteins such as nephrin, CD2-associated protein (CD2AP), zonula occludens-1(ZO-1), and transient receptor potential channel-6 (TRPC-6) et al., that ensure the stable anchorage of the membrane proteins of the slit diaphragm to the actin cytoskeleton in the podocyte (22, 23). These membrane multi-protein complexes localizing on the slit diaphragm in podocytes are capable of regulating the mechanosensation and cell signaling to maintain the integrity of the glomerular filtration barrier (23). The loss of the exon 5-coded central part, an essential component of the prohibitin homology domain, results in the podocin's inability to bind the cholesterol of the inner membrane of the cell (20) and abnormal distribution of podocyte-associated molecules (24). Nephrin is one of the most important components of the slit diaphragm in the podocyte, and nephrin exerts its role as the signal transducer through the phosphorylation of AP-1. The nephrin-activated AP-1 is augmented by podocin which is markedly attenuated by deletion of the prohibitin homology domain of podocin, thereby impairing the function of nephrin in podocytes (24, 25). Intriguingly, how does the replacement of glutamic acid in the 198th position of the amino acid sequence with the alanine lead to a significant loss of podocin function in podocytes, as observed in the family we described in this study, remains to be further determined.

SRNS caused by *NPHS2* mutation is believed to be a recessive inheritance disease requiring a mutation on both alleles coding for podocin, and these patients usually progress to an early onset of ESKD, such as the female patient reported here who started on hemodialysis at the age of 14 years old. Hence, it is very essential to adopt an appropriate therapeutic approach for these patients. Apart from the dialysis, burgeoning evidence has been furnished that renal transplantation may favor ESKD patients with genetic SRNS. In 2020, the results of 101 cases of kidney transplantation in children with SRNS in Italy reported that no disease recurrence was observed in all 41 genetic SRNS including *NPHS2* mutation compared to the idiopathic SRNS (59.5% recurrence) or unknown reasons SRNS patients (43.5% recurrence), during a median follow-up of 58.5 months (26). A cohort study in Germany involving 27 multiplex families (53 patients) and 25 patients with sporadic SRNS due to the *NPHS2* mutation showed these patients who underwent kidney transplantation did not develop recurrence of proteinuria (27). In the meantime, few reports suggested a risk of relapse in the SRNS due to *NPHS2* mutation (28, 29), however. the causative role of the *NPHS2* variants in these reports might be reconsidered with a further in-depth understanding of the pathogenic *NPHS2* variants in the SRNS; no anti-podocin antibodies have been detected in the tested *NPHS2* patients with disease recurrence after kidney transplantation (28, 30); many patients with disease recurrence showed an effective response to immunosuppression treatment (28, 29). The female patient with ESKD in this study has been recommended for kidney transplantation.

In conclusion, this paper reports an SRNS phenotype of familial NS with a pathological lesion of FSGS. Although the exact mechanism remains to be clarified, we offer evidence that the homozygous exon 5 c.593 A>C (p. E198A) of the *NPHS2* gene plays a significant role in proper podocin protein function and its pathogenesis. The nephrologists are encouraged to do the genetic testing for these early-onset SRNS patients especially those with a positive family history, as it helps in their right diagnosis, proposes the appropriate therapeutic strategies, predicts the prognosis, and assists genetic and reproductive counseling.

## Data Availability Statement

The datasets presented in this study can be found in online repositories. The names of the repository/repositories and accession number(s) can be found below: The National Omics Data Encyclopedia (NODE), accession number: OEP002338.

## Ethics Statement

The studies involving human participants were reviewed and approved by Medical Ethics Committee of People's Hospital of Xinjiang Uygur Autonomous Region. Written informed consent to participate in this study was provided by the participants' legal guardian/next of kin. Written informed consent was obtained from the individual(s), and minor(s)' legal guardian/next of kin, for the publication of any potentially identifiable images or data included in this article.

## Author Contributions

LB, JZ, and HJ made clinical data collection and were actively involved in the clinical care of the patients. XT, CL, and HJ made substantial contributions to the conception of the manuscript. HJ and CZ evaluated the renal pathology of the patients. XT, LB, and HJ drafted and edited the manuscript. All authors read and approved the final manuscript.

## Funding

This work was supported by the Xinjiang Uygur Autonomous Region Project Application of bone disease information processing system in end-stage kidney disease (2020E02118) to HJ.

## Conflict of Interest

The authors declare that the research was conducted in the absence of any commercial or financial relationships that could be construed as a potential conflict of interest.

## Publisher's Note

All claims expressed in this article are solely those of the authors and do not necessarily represent those of their affiliated organizations, or those of the publisher, the editors and the reviewers. Any product that may be evaluated in this article, or claim that may be made by its manufacturer, is not guaranteed or endorsed by the publisher.
